# Longitudinally Extensive Transverse Myelitis in a Patient With Systemic Lupus Erythematosus, Recent Influenza Infection, and Nutritional Deficiencies in the Setting of Chronic Alcohol Use

**DOI:** 10.7759/cureus.61601

**Published:** 2024-06-03

**Authors:** Jesus R Salas, Anna Farrell, Casey Potts, Peter Robinson

**Affiliations:** 1 Neurology, The Ohio State University Wexner Medical Center, Columbus, USA; 2 Internal Medicine, The Ohio State University College of Medicine, Columbus, USA

**Keywords:** neurology of systemic disease, nutritional neuropathy, inflammatory myelitis, neuropsychiatric systemic lupus erythematosus, longitudinally extensive transverse myelitis

## Abstract

Longitudinally extensive transverse myelitis (LETM) is traditionally classified as an inflammatory disorder of the spinal cord spanning three or more vertebral segments. The differential diagnosis for TM is vast and can include infectious, nutritional, and can even be idiopathic in some reported cases. However, autoimmune etiologies such as systemic lupus erythematosus (SLE) can rarely present with neurological manifestations such as LETM. In this case report, we present a 33-year-old female with a prior history of SLE who developed an LETM in the setting of possible provoking factors such as nutritional deficiencies and a recent viral illness. In this case report, we highlight her clinical course, recovery, and working differential diagnosis after laboratory testing and neurological imaging. Finally, we discuss the different treatments that ultimately lead to her successful recovery after her prolonged clinical course.

## Introduction

Transverse myelitis (TM) is an acquired inflammatory disorder of the spinal cord usually presenting with rapid onset weakness, sensory deficits, and bowel/bladder dysfunction. This condition often occurs as a complication of infection. However, it is important to note that TM may also exist as part of a continuum of neuro-inflammatory disorders [1].

TM occurs in the spinal cord at any level, but most commonly affects the thoracic region and can affect several vertebral segments [1]. However, longitudinally extensive transverse myelitis (LETM) is a rarer form of TM, often leading to widespread inflammation of the spinal cord traditionally extending across three or more vertebral segments. The disorder transverses the spinal cord causing bilateral neurological deficits. There may only be partial or asymmetric involvement. The duration of this disease may be as little as 3 to 6 months or may become permanently debilitating. At peak deficit with acute TM, 50% of patients are completely paraplegic with virtually all of the patients having some degree of bladder/bowel dysfunction. Of patients who develop TM, 33% of patients recover with little to no lasting deficits, 33% have a moderate degree of permanent disability, and 33% are permanently disabled [1].

The incidence of TM is approximately 1-8 new cases per 1 million people per year [1-2]. In one case series, 64% of cases were idiopathic (primary TM) and 36% were associated with a disease (secondary TM). Other reports include idiopathic TM accounting for 15-30% of cases [2]. This disorder can affect men and women equally and can affect patients of all ages, however, there is a bimodal peak between ages 10-19 and 30-39 [3,4]. It is important to note some predilection based on certain epidemiological factors. One example is that women tend to predominate presentations with TM in the setting of multiple sclerosis. While approximately one-third of patients with TM recover with little to no deficit, reportedly 33,000 Americans are left with a moderate to severe degree of disability [4].

There are several possible etiologies of TM, but they can be broadly divided into idiopathic, postinfectious, systemic inflammation, or multifocal central nervous system disease. The following case report presents an interesting case of a 33-year-old female with several risk factors who developed LETM over several weeks. Our patient underwent an extensive biochemical and radiological workup, leading to successful treatment and minimal deficits after following up in the clinic several months after her initial presentation.

## Case presentation

Presentation and admission****

A 33-year-old Caucasian female with a significant past medical history of systemic lupus erythematosus (SLE), depression, anxiety, tobacco use disorder, and alcohol abuse disorder (per patient approximately 750 ml of tequila daily for the past several years). As mentioned previously, the patient was initially diagnosed with SLE in 2013 after presenting with vitiligo, arthralgias, and joint effusions. Serologically she was antinuclear antibody (ANA) positive, dsDNA positive, C3 low, C4 low, and without reported organ involvement (no renal involvement). She was treated with maintenance daily hydroxychloroquine 200 mg and belimumab injections which had only been obtained once prior to her presentation. 

**Figure 1:**
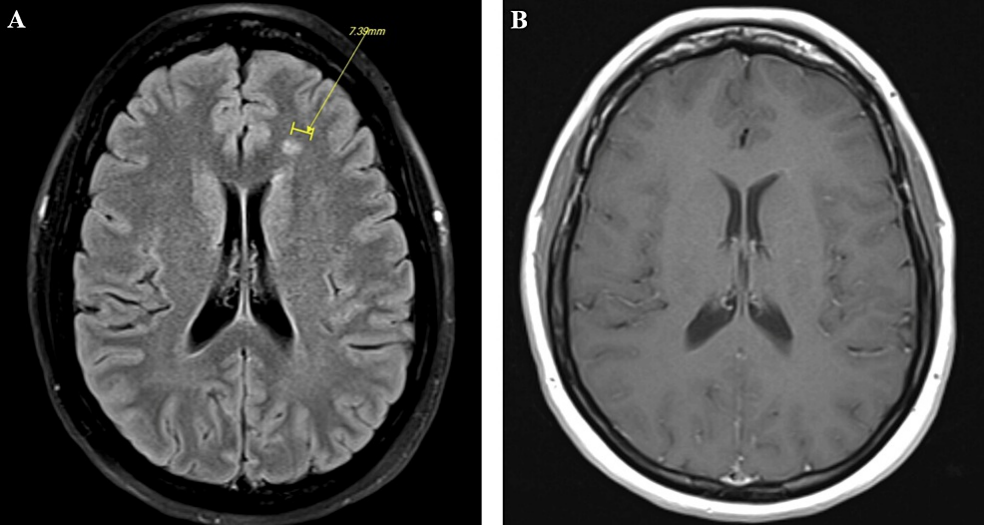
MRI of the brain with and without contrast. A) MRI of brain T2 fluid-attenuated inversion recovery (FLAIR) axial cut; B) MRI of brain T1 post-contrast axial cut Diffuse abnormal hyperintensity appreciated specifically a small T2 hyperintense foci in the deep white matter of the left frontal lobe, superior to the left frontal horn with no contrast enhancement seen on post-contrast imaging.

**Figure 2:**
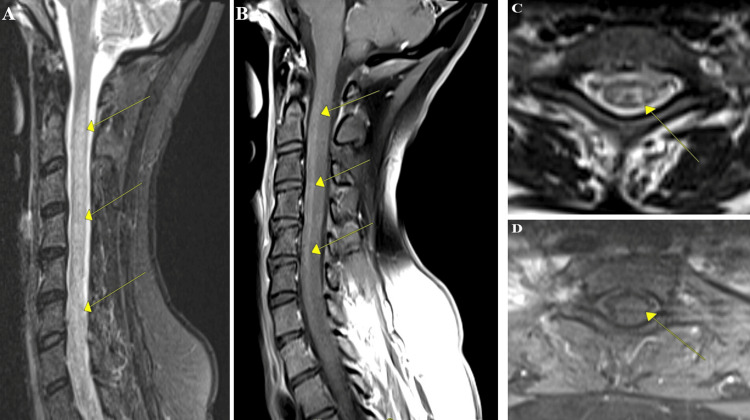
MRI of the cervical spine with and without contrast. A-B) Diffuse and extensive T2 hyperintensities in the cervical and upper thoracic spine with mild contrast enhancement as demonstrated by the yellow arrows on sagittal cuts appreciated; C-D) axial MRI images at C6 with T2 hyperintensities with mild contrast enhancement shown with the yellow arrows.

**Figure 3:**
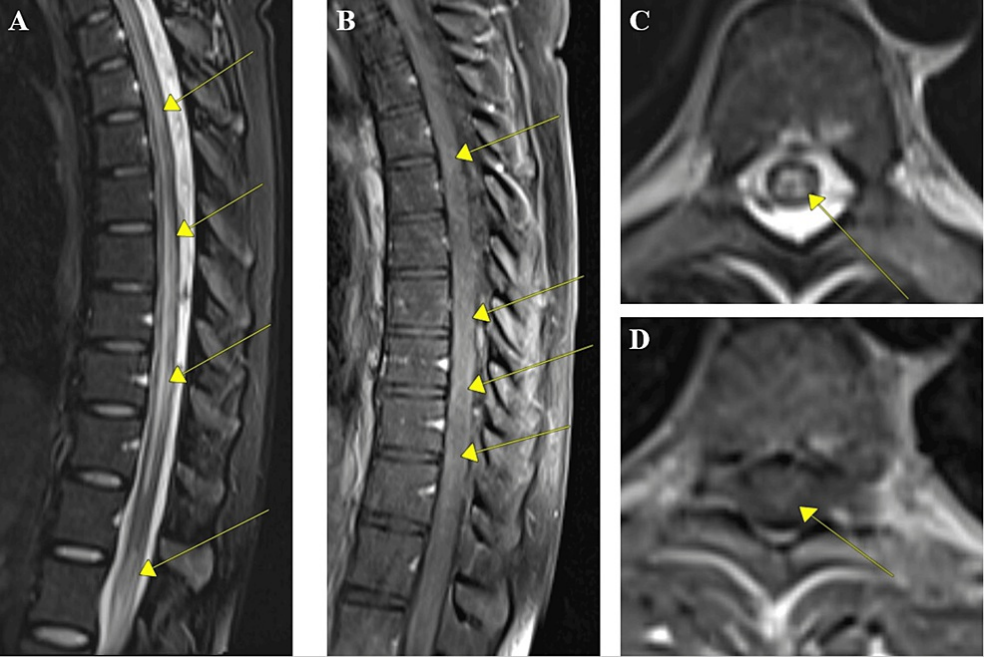
MRI of the thoracic spine with and without contrast. Diffuse abnormal T2 hyperintensity through the spinal cord from the lower cervical spine to T11 and in the distal conus medullaris. A-B) MRI of thoracic spine sagittal shows several longitudinally extensive lesions with mild pathological contrast enhancement. C-D) Axial MRI of thoracic spine imaging at T8 with hyperintensity and mild contrast enhancement.

**Figure 4:**
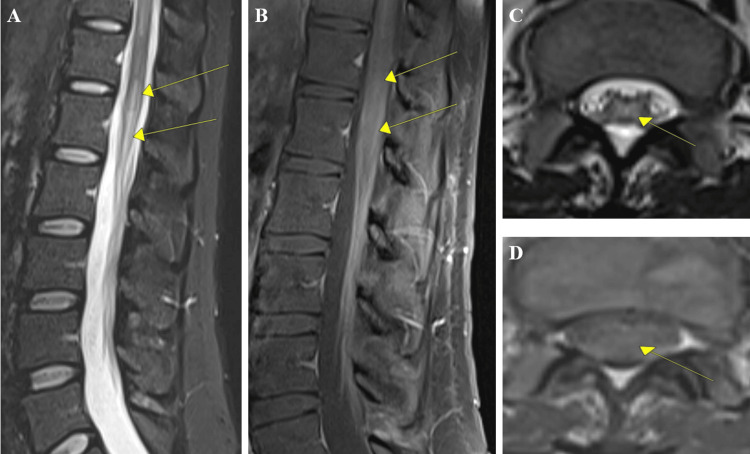
MRI of the lumbar spine with and without contrast. A-B) MRI of lumbar spine abnormal T2 hyperintensities in the spinal cord and distal conus medullaris with mild pathological contrast enhancement of the conus medullaris and the cauda equina. C-D) Axial cuts with T2 hyperintensity of the conus medullaris with mild contrast enchantment.

On presentation to the outside hospital, the patient had experienced 3 days of progressive bilateral lower extremity weakness rendering her unable to walk. She had urinary and bowel incontinence that progressively worsened. During the patient’s initial hospitalization, her symptoms progressed to the point that she did not have a bowel movement for more than 3 days. She also required Foley catheter placement given persistent urinary retention. At that time, she developed fevers, fatigue, chills, and myalgias. She was discovered to be positive for influenza A and negative for SARS-CoV-19. Given the progression of her symptoms, MRI scans of the brain, cervical, thoracic, and lumbar spine were obtained with and without contrast. Findings of imaging revealed a nonspecific small T2 hyperintense foci in the deep white matter of the left frontal lobe, possibly as a result of chronic microvascular changes or a demyelinating process. Diffuse abnormal T2 hyperintensity throughout the spinal cord from the upper cervical spine down to T11 and in the distal conus medullaris with mild enlargement of the spinal cord at the conus was also appreciated. Notably, there was mild pathologic contrast enhancement of the spinal cord in the regions correlating with abnormal T2 hyperintensity. The results of imaging can be found in Figures 1-4.

After the patient had experienced 1 week of symptoms, she was transferred to our tertiary medical center. Vital signs on evaluation, BP 130/80, heart rate 50, respiration 16, temperature of 36.7°C. Physical examination was pertinent for the following neurological deficits; right lower extremity strength 4/5, left lower extremity strength 3/5, bilateral Babinski reflex mute, dysesthesias, and loss of sensation to pin-prick appreciated in the L1, L2, L3, L4 distribution bilaterally, mild saddle anesthesia (S3, S4, S5) also was appreciated otherwise sensation was intact above these spinal levels. Gait examination unable to be formally assessed given her profound weakness.  Recurrent bladder scans with urinary retention greater than 800 cc of urine. A Foley catheter was continued for urinary retention during admission and at discharge.

Serum laboratory values obtained during admission are shown in Table 1. Her complete blood count (CBC), liver function tests (LFT), calcium, magnesium, phosphorus, and chem 7 basic metabolic panel (CHM7) on presentation were unremarkable. She was noted to have elevated anti-cardiolipin IgM; however, anti-cardiolipin IgG was negative. C3 and C4 were observed to be on the low end of normal. Consistent with her prior history of SLE, she was ANA positive, Sjögren’s-Syndrome-related antigen A (SSA) positive, and had elevated dsDNA titers. The patient was also noted to have several nutritional deficiencies. Vitamin B12 and B6 were on the low end of normal. Methylmalonic acid (MMA) was not obtained. Consistent with her history of alcohol use, her vitamin B1 level was undetectable (< 2 nmol/L). Her copper and zinc levels were also discovered to be low. During her workup, she was discovered to have vitamin D deficiency with a level of 16.4 ng/mL. Lastly, additional autoimmune culprits of LETM were investigated such as myelin oligodendrocyte glycoprotein (MOG) antibody, neuromyelitis optica (NMO)/AQP4 IgG ELISA, HIV, and syphilis/rapid plasma reagin (RPR). These laboratory tests were all negative.

**Table 1:** Serum laboratory values ANA: antinuclear antibody; MOG: myelin oligodendrocyte glycoprotein; NMO: neuromyelitis optica; dsDNA: double-stranded DNA; anti-SSA: anti-Sjögren’s-syndrome-related antigen A; RPR: rapid plasma reagin. Chem 7 and CBC were unremarkable at the time of presentation and not included.

Lab	Patient Value	Normal Value
MCV	98.5 fL	79.6-97.7 fL
Albumin	2.7 g/dL	3.5-5.0 g/dL
Calcium	9.0 mg/dL	8.6-10.5 mg/dL
Lupus anticoagulant	Negative	
Anti-cardiolipin IgM	30.8 CU	< 10.0
Anti-cardiolipin IgG	Negative	< 10.0
Vitamin B12	308 pg/mL	211-911 pg/mL
Vitamin B6	10 mcg/L	5-50 mcg/L
Vitamin B1	< 2 nmol/L	4-15 nmol/L
Vitamin D	16.4 ng/mL	30.0-100.0 ng/mL
Vitamin E	9.3	5.5–17
Copper	70 mcg/dL	77-206 mcg/dL
Zinc	62 mcg/dL	60-106 mcg/dL
MOG antibody	Negative	
NMO/AQP4 IgG ELISA	Negative	
HIV	Negative	
Syphilis/RPR	Negative	
ANA	Positive	
SSA	Positive	
dsDNA	105	< 4 I/U
C3	84 mg/dL	87-200 mg/dL
C4	12 mg/dL	18-52 mg/dL
Influenza A	Positive	
SARS-CoV-19	Negative	

The patient underwent a lumbar puncture with an opening pressure of 11 cmH2O. CSF studies are shown in Table 2. Of note, she had mild pleocytosis with a CSF cell count of 21 nucleated cells, with a differential revealing 99% lymphocytes. The patient’s CSF protein and glucose were within normal limits. There were no unique oligoclonal bands identified in the CSF. The remainder of her CSF studies were unremarkable.

**Table 2:** Cerebral spinal fluid laboratory results ACE: angiotensin-converting enzyme

Lab	Patient Value	Normal Value
Glucose	48	40-70 mg/dL
Protein	34	15-45 mg/dL
WBC	21 (99% lymphocytes)	< 3
RBC	23	< 3
Oligoclonal bands	0	< 2
Meningitis/encephalitis panel	Negative	
Paraneoplastic panel	Negative	
ACE	1.4 U/L	0.0-2.5 U/L

Given the patient’s complex presentation, she was started on IV Solu-Medrol 1000 mg for 5 days. She was then transitioned to prednisone 60 mg daily with a plan of decreasing by 10 mg every 2 weeks. Given her prolonged steroid taper, she continued calcium and vitamin D supplementation, famotidine, and DS Bactrim for *Pneumocystis jirovecii* pneumonia (PJP) prophylaxis. During this period, the patient was instructed to continue hydroxychloroquine 200 mg daily until evaluation in the neurology and rheumatology clinic. Given concern for a nutritional component contributing to her presentation, her copper, zinc, vitamin D, vitamin B12, and thiamine were appropriately replaced during admission and at discharge. Over a 3-week period, she continued to improve slowly and was discharged to in-patient rehabilitation (IPR). Given her clinical course during admission, plasmapheresis was deferred. At the time of discharge from IPR, she was independent in self-care, transfers, and mobility with an assistive device, however, she still experienced urinary retention.

Post-hospital evaluation

The patient followed up with rheumatology and neurology at 3 and 6 months after discharge, respectively. Neurologically she had no focal deficits and was able to ambulate without difficulty. Her exam was only notable for 3+ reflexes in the bilateral patella and Achilles tendons, however, was still experiencing urinary and bowel symptoms consistent with neurogenic bladder and bowel. She had been following up with urology and was considered a candidate for a spinal stimulator. She was continued on hydroxychloroquine 200 mg daily and was started on rituximab 1 g IV twice separated by 2 weeks after discharge and then repeated every 6 months thereafter. On repeat laboratory testing at follow-up, C4 was still noted to be low at 14 mg/dL and C3 was normal at 110 mg/dL. The patient’s B12 remained on the low end of normal at 372, and copper levels had increased to 123 mcg/dL at the 3-month follow-up visit.

Due to her prior history of SLE and extensive cord involvement, the findings ultimately favored lupus-related myelitis rather than postinfectious immune-mediated inflammatory disorder. However, given her history of extensive alcohol use, it was suspected that her nutritional deficiencies also likely contributed to her presentation.

## Discussion

In this case, we report a 33-year-old female who presented with an LETM in the setting of multiple risk factors including a relevant history of SLE, nutritional deficiencies, and recent influenza infection. Our patient recovered after vitamin supplementation, 5 days of 1 g IV methylprednisolone with oral prednisone taper, and 1 g IV rituximab induction twice over a period of 2 weeks and then continued rituximab maintenance 1 g IV every 6 months for maintenance therapy. One year after her presentation and extensive physical therapy, she can ambulate and only has intermittent urinary incontinence which she has closely followed with urology.

In our case report, the patient’s prior history of SLE was of concern as she had a history of some systemic involvement in the past during acute flares (arthralgias and skin changes). SLE manifestations can involve the central nervous system, specifically the spinal cord although uncommon. The prevalence of neurological and psychiatric involvement in SLE is variably reported in the literature ranging from 25 to 75%, with a higher incidence being noted in patients with early lifetime diagnoses [5]. Higher mortality in neuropsychiatric lupus is associated with the following risk factors including antiphospholipid antibodies, young age, greater disease activity, and organ damage. Specifically, SLE-induced myelitis commonly involves three or more vertebral levels. Retrospective studies have highlighted that patients with SLE and LETM have inflammation of both gray and white matter of the spinal cord. Those with gray matter lesions typically presented with lower motor neuron signs of weakness, flaccid paralysis, and hyporeflexia compared to white matter involvement. In our case report, gray matter appeared to be most pronounced as the patient had bilateral leg weakness, hyporeflexia as well as urinary retention [6]. This is also seen on the MRI axial cuts through the spinal cord as the hyperintensities appear to be located in the matter.

Given our patient’s extensive alcohol history, she was noted to have several nutritional deficiencies specifically B12 and copper which can present with an LETM. Unfortunately, MMA was not obtained and thus we were unable to fully conclude if B12 deficiency truly was a contributing factor to her presentation. A typical neurological picture of copper deficiency is ataxic myelopathy, unsteady gait with mixed features of sensory ataxia, and spasticity produced by posterior and lateral column dysfunction [7]. The clinical and radiological features of copper deﬁciency closely resemble those seen in vitamin B12 (cobalamin) deﬁciency and both can even co-exist in the same patient [7]. Vitamin B12 deficiency classically presents with loss of vibration and proprioception in the hands and feet, with eventual progression to sensory loss of all modalities, sensory gait ataxia, and distal muscle weakness, especially in the lower extremities. It is important to note that a patient may develop pyramidal dysfunction leading to subacute combined degeneration. In this patient’s presentation, it is quite possible that nutritional deficiencies contributed to her symptoms given her history of malnutrition and alcohol use.

Prior to the patient’s presentation to our medical center, she experienced an infection with influenza A. In one case report by Márquez and Vilá, a 30-year-old patient with SLE developed a longitudinally TM after an influenza A infection [8]. The case report by Márquez and Vilá presents a patient with urinary retention, bilateral lower extremity paralysis, upper extremity weakness, and ocular involvement with optic nerve inflammation and macular edema. Specifically, hypocomplementemia was appreciated, with low C3 and C4. MR imaging showed C4-T12 hyperintense lesions consistent with TM. In our case report, our patient experienced a similar cadence of events, which may have led to a proinflammatory state precipitating her LETM, or at the minimum contributed to her presentation.

Treatment of neuropsychiatric manifestations of SLE

The treatment for neurological manifestations of SLE revolves around glucocorticosteroids and cyclophosphamide. An accepted practice consists of 1 g of methylprednisolone intravenously for 3 consecutive days followed by prednisolone orally (starting dose 1 mg/kg/day), with a tapering scheme over 3 to 12 months. Cyclophosphamide induction regimens consist of 500 to 750 mg/m^2^/month. After 6 months of therapy, cyclophosphamide can be continued in the same dose every 3 months for 18 months, or maintenance therapy during 1 year with other immunosuppressive therapies [9]. In our case report, rituximab was chosen as maintenance therapy. Khabbazi et al. presented a case report in which a patient presenting with SLE-induced myelitis was successfully treated with rituximab 1 g two times with an interval of 2 weeks, with the patient having some neurological improvement after a period of 6 months [10-14]. It is important to note that rituximab is used only off-label for SLE neuropsychiatric management refractory to multiple medications due to insignificant randomized control trial results. It may be unclear clinically whether CNS manifestations of SLE are either embolic, inflammatory, or both in nature. In these cases, glucocorticoids or immunosuppressive agents may be given in combination with antiplatelets or anticoagulation. Similarly, inflammatory etiologies of LETM are treated with glucocorticosteroids.

Vitamin and mineral deficiencies are also treated. In our case report, vitamin B12 was replaced by oral supplementation of 1000 mcg daily. There are no clear guidelines for monitoring and repeating B12 levels in the medical literature [10]. In our case, B12 levels were repeated in 3 months with slight improvement from 308 to 372 pg/mL. MMA was not obtained on repeat evaluation, and thus we cannot fully conclude that a true B12 deficiency was present. Copper was additionally supplemented with a daily multivitamin. As per the guidelines set by the NIH, the daily requirement of copper for an adult female older than 19 years old is 900 mcg per day [11]. After 3 months of supplementation, the patient’s copper level increased from 70 to 123 mcg/dL.

Lastly, it is important to discuss symptomatic treatment for neuropathic pain due to LETM. Often gabapentin or pregabalin are utilized for hyperesthesia experienced by patients. Additionally, serotonin-norepinephrine reuptake inhibitors such as duloxetine, have been utilized for neuropathic pain. Studies show that duloxetine inhibits 5-HT and norepinephrine uptake in the hypothalamic synaptosomes, leading to the augmentation of pain in the central nervous system [12,13]. Given our patient’s dysesthesias, she was trialed on gabapentin 300 mg TID (three times a day) and escitalopram 10 mg QHS (every night at bedtime) for her symptoms. At follow-up, she was only compliant with gabapentin with some control of her symptoms.

## Conclusions

LETM can have a variety of etiologies and the differential diagnosis may be broad when first encountering a patient presenting with this unique neurological syndrome. In some cases of TM, a likely etiology can be identified with an appropriate history and physical exam with accompanying biochemical testing and radiologic imaging. However, it is additionally equally important to consider a multifactorial etiology of TM as is seen in this case report. All differential diagnoses should be considered. Any identifiable inflammatory, vascular, or biochemical etiologies should be treated in accordance with evidence-based medicine. Patients presenting with this neurological syndrome should have close follow-up and evaluation with a multidisciplinary team of neurologists, rheumatologists, urologists, physical medicine, and rehabilitation physicians to improve their chances of successful recovery with minimal deficits and a high level of independent functioning.
